# Fuzzy System Based Medical Image Processing for Brain Disease Prediction

**DOI:** 10.3389/fnins.2021.714318

**Published:** 2021-07-30

**Authors:** Mandong Hu, Yi Zhong, Shuxuan Xie, Haibin Lv, Zhihan Lv

**Affiliations:** ^1^College of Computer Science and Technology, Qingdao University, Qingdao, China; ^2^North China Sea Offshore Engineering Survey Institute, Ministry of Natural Resources North Sea Bureau, Qingdao, China

**Keywords:** fuzzy system, brain image, digital twins, image segmentation, fuzzy clustering, HPU-Net

## Abstract

The present work aims to explore the performance of fuzzy system-based medical image processing for predicting the brain disease. The imaging mechanism of NMR (Nuclear Magnetic Resonance) and the complexity of human brain tissues cause the brain MRI (Magnetic Resonance Imaging) images to present varying degrees of noise, weak boundaries, and artifacts. Hence, improvements are made over the fuzzy clustering algorithm. A brain image processing and brain disease diagnosis prediction model is designed based on improved fuzzy clustering and HPU-Net (Hybrid Pyramid U-Net Model for Brain Tumor Segmentation) to ensure the model safety performance. Brain MRI images collected from a Hospital, are employed in simulation experiments to validate the performance of the proposed algorithm. Moreover, CNN (Convolutional Neural Network), RNN (Recurrent Neural Network), FCM (Fuzzy C-Means), LDCFCM (Local Density Clustering Fuzzy C-Means), and AFCM (Adaptive Fuzzy C-Means) are included in simulation experiments for performance comparison. Results demonstrate that the proposed algorithm has more nodes, lower energy consumption, and more stable changes than other models under the same conditions. Regarding the overall network performance, the proposed algorithm can complete the data transmission tasks the fastest, basically maintaining at about 4.5 s on average, which performs remarkably better than other models. A further prediction performance analysis reveals that the proposed algorithm provides the highest prediction accuracy for the Whole Tumor under DSC (Dice Similarity Coefficient), reaching 0.936. Besides, its Jaccard coefficient is 0.845, proving its superior segmentation accuracy over other models. In a word, the proposed algorithm can provide higher accuracy, a more apparent denoising effect, and the best segmentation and recognition effect than other models while ensuring energy consumption. The results can provide an experimental basis for the feature recognition and predictive diagnosis of brain images.

## Introduction

Now that economy develops unprecedentedly, health has become the top priority that affects people’s living standards. As a common disease that affects human health, brain diseases are classified based on their severity. Brain tumors are undoubtedly the brain diseases that pose the greatest threat to human health. Brain tissue is a functional organization with complicated structure and irregular shape. The accurate segmentation of brain images provides rich and valuable information for clinical surgeries. Nevertheless, medical images of brain tissues are usually susceptible to interference, such as noises, uneven grayscale, local volume effects, and artifacts (Jiang et al., 2019; Senthilkumar and Gnanamurthy, 2019). In the meantime, there are great difficulties in recognizing and segmenting brain images due to the weak contrast in the image edges and the complicated brain tissue structure.

AI (Artificial Intelligence) technologies, such as DL (Deep Learning), BD (Big Data), and IoT (Internet of Things), have been universally accepted in various fields; in that case, intelligent development in the medical field gets increasingly vital for monitoring people’s health status. Correct processing of medical images interfered by strong noises and geometric distortions is significant in clinical diagnosis and treatment (Zheng et al., 2020). DL approaches have made significant progress in image resolution recognition and segmentation; nevertheless, real medical images with low resolution are complicated and unknown. Influences of blurring factors, such as weakening of the image boundary and uneven grayscale, are extremely important. Since DNN (Deep Neural Network) does not consider the blurring while training the data, directly applying medical images processed by DL models to real-life settings often results in unideal reconstruction effects and even apparent blurs and artifacts (Pham et al., 2018; Mishro et al., 2020). The fuzzy theory breaks the traditional “one or the other” view, making the image information more thoroughly applied. Meanwhile, the fuzzy system, an unsupervised classification model, does not require manual intervention, whose segmentation process is completely automatic (Li et al., 2020). The uncertainty and ambiguity in the image can be processed well. Thus, correct processing of brain images can have rather practical significance for diagnosing and treating the brain diseases.

The imaging mechanism of NMR (Nuclear Magnetic Resonance) and the complexity of human brain tissues cause the MRI (Magnetic Resonance Imaging) images to present varying degrees of noise, weak boundaries, and artifacts. Therefore, brain imaging processing for brain disease prediction provides extremely significant value for reducing the risks of clinical treatment. The innovative points are the improvements over fuzzy clustering regarding the noises, weak boundaries, and artifacts in brain images, and the brain image processing and brain disease diagnosis prediction model designed based on HPU-Net (Hybrid Pyramid U-Net Model for Brain Tumor Segmentation) and improved fuzzy clustering, to provide some theoretical support for feature recognition and prediction diagnosis based on brain images.

## Recent Works

### Existing Applications of Fuzzy System

The traditional “one or the other” idea in various fields cannot explain some actual situations. In contrast, the fuzzy logic system can break this traditional idea and is very useful in solving problems in actual applications. Many scholars have explored fuzzy systems. Feng and Chen (2018) combined TS (Takagi-Sugeno) fuzzy system with fuzzy BLS (Broad Learning System) and proposed a new neuro-fuzzy model and fuzzy BLS. In the meantime, they combined the defuzzification output of each fuzzy subsystem with the output of the enhancement layer to get the model output. Additionally, they employed K-Means to determine the center of the Gaussian membership function in the antecedent and the number of fuzzy rules. Eventually, they found that the performance of the fuzzy BLS model was better than that of other models (Feng and Chen, 2018). IT2 (Interval Type-2) fuzzy sampling data H∞ had parameter uncertainty, data loss, and transmission delay; to solve these problems, Du et al. (2019) connected the IT2 fuzzy system describing the network control system and the IT2 network sampling controller implemented by the zero-order holder in a closed-loop system. Then, they adopted the input delay approach to convert the closed-loop system into a continuous time-delay system. Simultaneously, they utilized continuous-time Lyapunov-Krasovskii functional theory for stability analysis. Eventually, the effectiveness and superiority of the designed method were validated through four different actual systems (Du et al., 2019). To promote the exchange of fuzzy systems between different programming systems, Xie et al. (2020) imported/exported fuzzy systems for the operability and practicality of the standard. They provided a case study to illustrate the advantages of fuzzy systems (Xie et al., 2020). Harifi et al. (2020) proposed an ANFIS (Adaptive Neuro-Fuzzy Inference System) based on the EPC (Emperor Penguins Colony) algorithm regarding the unresolved fuzzy status in biological systems or physical systems in nature. Then, on the benchmark dataset, they compared the optimized ANFIS with other non-derivative algorithms. Afterward, this algorithm was applied to solve the classic inverted pendulum problem. Results demonstrated that ANFIS based on EPC provided smaller errors and better performance than other algorithms in the training and testing stages (Harifi et al., 2020).

### Current Development of Medical Image Processing

Medical image segmentation is a vital tool for current clinical applications. It is also the backbone of many clinical diagnostic approaches, tumor treatments, and computer-integrated surgeries. Many scholars have researched this issue. Bello et al. (2019) practiced an FCN (Fully Convolutional Network) trained prior to anatomical shape to create a time-resolved 3D segmentation for cardiac image sequences obtained from cardiac MRI. Moreover, they verified model’s prediction accuracy, and how to utilize high-dimensional medical image data for complex computer vision tasks to effectively predict human survival was elaborated (Bello et al., 2019). Jeyaraj and Nadar (2019) put forward a new blocked DCNN (Deep Convolutional Neural Network) regarding the medical image of oral cancer. This network had a two-layer structure, which could mark and classify Regions of Interest (ROI) in multi-dimensional hyperspectral images. The classification accuracy of this network was compared with another traditional medical image classification algorithm. Results suggested that for complex medical images of oral cancer diagnosis, the proposed regression-based blocked DCNN could improve the diagnostic quality (Jeyaraj and Nadar, 2019). Renard et al. (2020) put forward an original overview of source variability to better understand DL medical image segmentation associated with challenges and problem reproducibility. In the end, they applied a DL framework to analyze different sources of variability for potential problems properly, and developed and verified an effective segmentation result evaluation system (Renard et al., 2020). Yoo et al. (2020) explored the feasibility of using DL decision tree classifiers to detect COVID-19 (Coronavirus Disease 2019) from CXR (Chest X-ray Radiography) images regarding the massive global outbreak of COVID-19. Compared with other domain models, the proposed DL-based decision tree classifier could pre-screen patients for classification and quick decision-making before the results of RT-PCR (Reverse Transcription Polymerase Chain Reaction) were available (Yoo et al., 2020).

To sum up, AI algorithms, such as DL, have been accepted in medical image processing; however, features of multiple boundary pixels are not continuous. Medical images with no apparent differences in pixel features on both sides of the boundary cannot be effectively processed. The fuzzy system algorithm is just an effective solution to no apparent difference in image boundaries. Thus, the fuzzy system is introduced based on DL algorithms to process medical images, which has great practical value for the accurate identification and diagnosis of brain images.

### Fuzzy System-Based Medical Image Processing for Brain Disease Prediction

Medical imaging equipment gets updated continuously as computer technology advances. Medical image segmentation is a vital link to medical image analysis, as well as a prerequisite and key technology for clinical surgeries. Nonetheless, due to individual differences and the complexity of human tissues such as the brain, the segmentation results of medical images, especially brain tumor images, are not satisfactory. As a result, countless researchers in this field worldwide have invested a lot of energies to research this issue.

### Demand of Applying Fuzzy System to Brain Image Diagnosis and Prediction

The amount of information in images keeps increasing with modern medical imaging technologies, making it necessary for medical workers to undertake a huge workload during medical image analysis. However, images appear to have low contrast, variability between tissues, the ambiguity between different tissues or between tissues and lesions due to the differences in the imaging principles of medical images and the characteristics of the tissues themselves (Barricelli et al., 2019; Kong et al., 2019). Therefore, the complexity and variety of medical images themselves have caused great obstacles to the accurate processing and analysis of medical images. Hence, using computer technology to assist brain image recognition and brain disease prediction is of vital significance for diagnosing and treating diseases.

**FIGURE 1:**
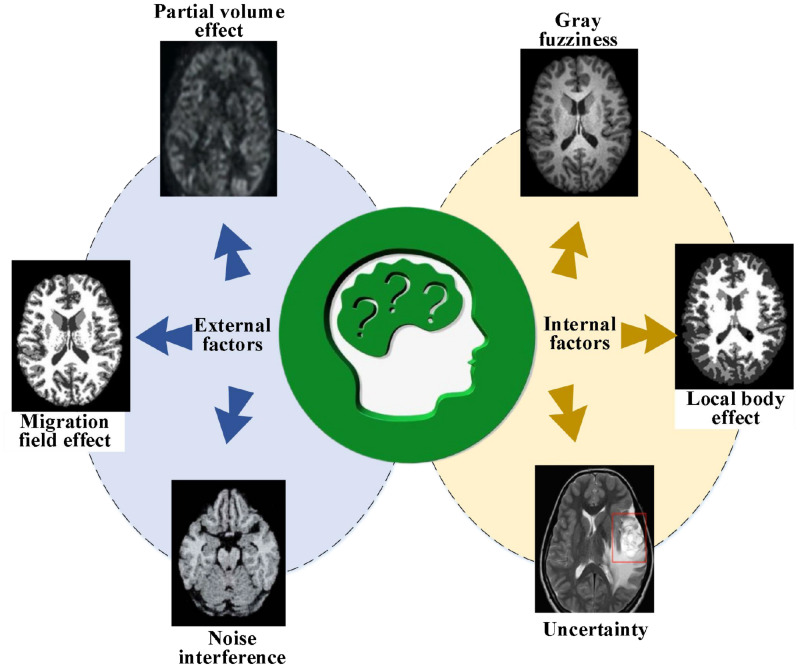
Problems in brain medical image processing.

Medical brain images have loads of uncertainty during diagnosis and prediction, and their essence lies in the ambiguity. There are three key manifestations. First, the grayscale is fuzzy: brain images are interfered with by lighting conditions and spatial resolution, making the edge of the pixel grayscale between the brain image boundary and the background blurry and overlap with each other. Second, it has a local body effect: due to the influence of equipment factors, voxels on a boundary often contain two substances: boundary and object, making it difficult to accurately describe the relationships among the edges, corners, and regions of the objects in the image. Third, there is uncertainty knowledge. In the case of pathological changes, brain images will have lumps or masses that are not available in normal tissues, bringing great difficulties in constructing the model. Besides, there may be noise interference, offset field effects, and partial volume effects in brain image processing. Figure 1 describes the problems of brain medical image processing.

Based on the above problems in brain medical image processing, this work uses the boundary information of unlabeled and labeled data in brain medical image as medical domain knowledge to further improve the effect of brain image segmentation and diagnosis. Moreover, the semi-supervised recognition method is introduced and deep learning algorithm is integrated based on semi-supervised learning. In different environments, the human brain MRI image is segmented. Finally, a brain disease medical image segmentation system is designed based on the improved algorithm.

### Segmentation Performance of Fuzzy System for Brain Images

The ambiguity of brain images refers to a characteristic between the brain medical image and the background. The uncertain boundary between the brain image and the background makes it difficult to segment such images because the machine will be affected by various factors during imaging. Actually, the most popular approach based on fuzzy theory is the FCM (Fuzzy C-Means) algorithm (Bai et al., 2018; Li et al., 2021). FCM not only avoids problems such as manually setting the threshold in advance but also has great advantages in dealing with the uncertainty and inaccuracy of images.

The evaluation indicators of cluster analysis are often judged based on the distance between the cluster centers, the number of samples in the cluster structure, and the standard deviation vector of the samples in the dataset and the cluster centers (Maqsood et al., 2020; Rahman et al., 2020; Wang Z. et al., 2020). The dataset *X* is divided into *C* categories, and *C* cluster sets *X_1_, X_2_,…, X_*C*_* are obtained. If the *C* cluster sets completely conform to Equation (1), it will be called the hard *C* division of *X*:

(1) $$\left\{ \begin{array}{c} X_{1}\cup X_{2}\cup \ldots \cup X_{c}=X\\ X_{i}\cap X_{k}=\emptyset ,1\leq i\neq k\leq c\\ X_{i}\neq \emptyset ,X_{i}\neq X,1\leq i\leq c \end{array}\right.$$

Fuzzy C division can determine the degree of uncertainty of each sample in the dataset in all classes. It can help to better understand and describe the features in the brain image. Regarding division results, fuzzy division can indicate the periphery of the division, the connection, and the dispersion between different division blocks. Hence, it can mine detailed information about the brain image (Liu et al., 2018). The core task of FCM is to minimize the weighted sum of squares of the distance from the classification sample point to each classification center. The weight is the membership function of each sample point to each classification center, which is the general criterion of the clustering algorithm (Ahmadi, 2020). The objective function of classification is obtained as (2):

(2) $$J_{\min}\,\left(U,V\right)=\sum_{i=1}^{c}J_{i}=\sum_{i=1}^{c}\sum_{j}^{n}u_{i\,j}^{m}\,d_{i\,j}^{2}$$

In (2), *U* refers to the classification matrix, *C* denotes the number of sample classes, which is determined based on actual situations,_*u_ij_*_ describes the corresponding sample membership function, *V* signifies the classification center vector,_*V* = _[_*_v_1_,v_2_,v_3_,…v_c_]_, d_ij_*_ refers to the distance from the *i*-th classification center to the *j*-th sample point, and *m* is a constant that should be determined before the algorithm runs. The difference between FCM and general clustering algorithm is that it carries out the weighted operation of membership degree. The constraint conditions described in Equation (3) can be obtained from the membership function:

(3) $$\sum_{i=1}^{c}u_{i\,j}=1,\forall j=1,\ldots ,n$$

According to Equations (2) and (3), the key process of FCM is to find the best combination of U and V so that *J(U,V)* can achieve the minimum value under constraint conditions. Therefore, Equation (4) can be obtained using the LaGrange multiplier:

(4) $$F=J(U,\upsilon_{1},...\upsilon_{c},\lambda_{1},...\lambda_{n})=\sum_{i=1}^{c}\sum_{j}^{n}u_{ij}^{m}d_{ij}^{2}+\sum_{j=1}^{n}\lambda_{j}(\sum_{i=1}^{c}u_{ij}-1)$$

In (4),_λ_j,j=1,2,…,n__ is the LaGrange operator. The first-order necessary optimization condition for the partial derivative of Equation (4) is:

(5) $$\frac{\partial F}{\partial \lambda }=\sum_{i=1}^{c}u_{i\,j}-1=0$$

(6) $$\frac{\partial F}{\partial u_{i\,j}}=m\,{\left(u_{i\,j}\right)}^{m-1}\,{\left(d_{i\,j}\right)}^{2}-\lambda =0$$

Finishing Equation (6) can obtain Equation (7):

(7) uij=[λm(dij)2]1(m−1)

Substituting Equation (7) into (3) can get Equation (8):

(8) ∑i=1cuij=∑i=1c(λm)1(m−1)[1(dij)2]1(m−1)=(λm)1(m−1){∑i=1c[1(dij)2]1(m−1)}=1

Thus, Equation (9) can be obtained:

(9) (λm)1(m−1)=1∑i=1c[λm(dij)2]1(m−1)

Substituting Equation (9) into (7) can get Equation (10):

(10) uij=1∑k=1c[dijdkj]2(m−1)

Because_*d_ij_*_ can be zero, two different cases should be considered, respectively. For_∀_j__, two sets_*I_j_*_ and *_I_j__* are defined as Equations (11) and (12), respectively:

(11) $$I_{j}=\left\{i|1\leq i\leq c,d_{i\,j}=0\right\}$$

(12) $${\tilde{I}}_{j}=\left\{1,2,\cdots ,c\right\}-I_{j}$$

Hence, the membership function that minimizes_*J_min_(U,V)*_ values:

(13) {uij=1∑k−1c[dijdkj]2(m−1)  Ij=ϕuij=0, ∀i∈ Ij, ∑i∈Ijuij=1 Ij≠ϕ

Similarly,_*J_min_(U,V)*_ is the smallest cluster center value. Then, the partial derivative of_*V_i_*_ is:

(14) $$\frac{\partial J}{\partial V_{i}}=\sum_{j=1}^{n}{\left(u_{i\,j}\right)}^{m}\,\frac{\partial }{\partial V_{i}}\,\left[{\left(x_{j}-V_{i}\right)}^{T}\,A\,\left(x_{j}-V_{i}\right)\right]=0$$

(15) $$\sum_{j=1}^{n}{\left(u_{i\,j}\right)}^{m}\,\left[-A\,\left(x_{j}-V_{i}\right)\right]=0$$

(16) $$\sum_{j=1}^{n}{\left(u_{i\,j}\right)}^{m}\,\left(x_{j}-V_{i}\right)=0$$

Eventually, Equation (17) can be obtained:

(17) $$V_{i}=\frac{\sum_{j=1}^{n}{\left(u_{i\,j}\right)}^{m}\,x_{j}}{\sum_{j=1}^{n}{\left(u_{i\,j}\right)}^{m}}$$

According to the number of cluster categories of the brain image data sample set and the prescribed fuzzy weight of the brain image sample, the cluster center and the best classification matrix can be determined by Equations (16) and (17). The objective function can be optimized by iteratively solving its minimum value.

Furthermore, the classic FCM gets improved. Spatial information constraints are introduced to increase the algorithm’s resistance to noise in brain images, and the objective function can be obtained:

(18) $$\begin{array}{l} J_{improved}(U,\nu_{1},...\nu_{c},\lambda_{1},...\lambda_{n})\\ =\sum_{i=1}^{c}\sum_{j=1}^{n}u_{ij}^{m}||x_{j}-V_{i}|{|}^{2}+\frac{\alpha }{N_{R}}\sum_{i=1}^{c}\sum_{j=1}^{n}u_{ij}^{m}{\sum_{r\in N_{j}}\left||x_{r}-V_{i}|\right|}^{2} \end{array}$$

The latter term in Equation (18) is the spatial constraint information, equivalent to the square error about element *i*, and its essence is similar to the mean filtering for point *i*. The noise removal or resistance is performed on the image similar to the nature of spatial filtering so that the algorithm has stronger anti-noise performance. Besides, the edge information of brain images should be enhanced so that the image will not become blurred while denoising. Traditional average filtering and median filtering cannot meet these requirements. Anisotropic diffusion filtering based on partial differential equations, that is, the PM (Perona and Malik) model, can remove the noises in the image while retaining or even enhancing the edge information. Concepts of mass diffusion theory and iterative smoothing are introduced into image processing (Zhang et al., 2020). The PM model is described as:

(19) $$\frac{\partial I}{\partial t}=d\,i\,v\,\left(c\cdot \nabla I\right)$$

In (19), *I* refers to the original pixel brightness signal, *t* refers to the diffusion time,_∇_ refers to the gradient calculation, *div* refers to the divergence calculation, and *c* is the diffusion coefficient of the algorithm, namely a function of the image element gradient, and its value range is [0, 1], defined as Equation (20):

(20) c=f⁢(|∇⁡I|)

The diffusion system determines how the anisotropic algorithm diffuses. An adaptive diffusion control strategy can be used; more diffusion operations can be performed where there is noise in the brain image, and the diffusion can be stopped in the edge area of the image to retain the edge information (Mahata and Sing, 2020). Two diffusion functions commonly used at present are:

(21) $$c_{1}=\exp\left(-{\left(\frac{\nabla_{i}I_{i}}{\lambda }\right)}^{2}\right)$$

In (21),_∇_i__ represents the gradient of direction *i*, and_λ_ represents the threshold of the gradient that determines how much the edge of the brain image can remain after being diffused.

(22) $$c_{2}=\frac{1}{1+{\left(\frac{\nabla_{i}I_{i}}{\lambda }\right)}^{1+\alpha }}$$

**FIGURE 2:**
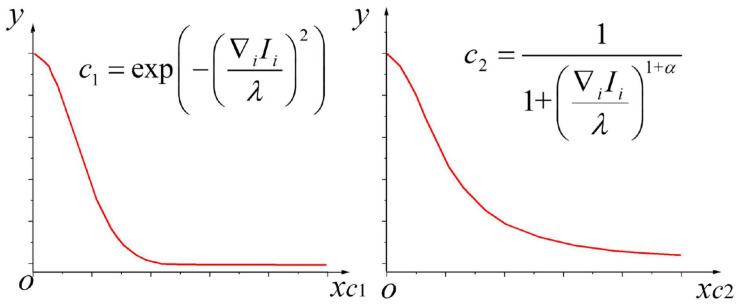
Characteristic curves of diffusion coefficients_*c_1_*_ and _*c_2_*_.

In (22),_α_ demonstrates the parameter of this equation, and_α∈0∞_. Here, the whole ${}_{\frac{\nabla_{i}I_{i}}{\lambda }}$ is taken as the independent variable of coefficient *c*. When_α=1_, the characteristic curves of diffusion coefficients_*c_1_*_ and _*c_2_*_ are demonstrated in Figure 2.

In these two types of commonly used diffusion functions, the gradient threshold plays a vital role during anisotropic diffusion called the diffusion constant (Zhao et al., 2018). The stream function is evaluated to clarify the relationship between diffusion constant_λ_ and diffusion function:

(23) φ⁢(x,t)=c⁢(x,t)⁢∇⁡I⁢(x,t)⁢|∇⁡I|<λ

(24) $$\frac{\partial I}{\partial t}=d\,i\,v\,\left(\varphi \,\left(x,t\right)\right)\,\left|\nabla I\right|>\lambda >1$$

**FIGURE 3:**
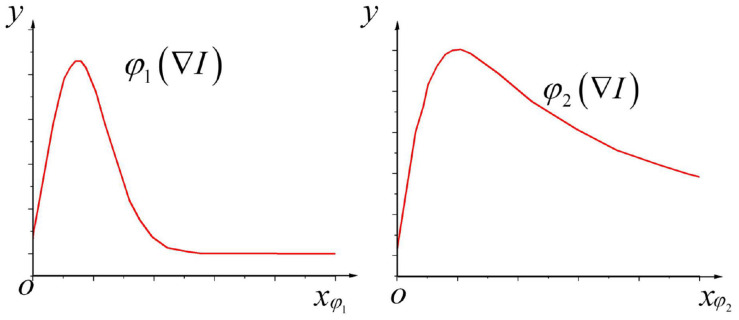
Characteristic curves of stream functions corresponding to diffusion coefficients_*c_1_*_ and _*c_2_*_.

Besides,_φ_1_(∇I),φ_2_(∇I)_ are defined as the stream functions corresponding to the above two types of diffusion functions; if_∇I/λ_ serves as the independent variable, characteristic curves of stream functions corresponding to these two diffusion coefficients when_α=1_ are demonstrated in Figure 3:

According to Figure 3, when the absolute value of the pixel gradient is_|∇I|<λ_; that is, when_|∇I|/λ<1_, the currently calculated element is not on the image edge. Simultaneously, as the image gradient increases continuously, the value of the stream function also increases, which increases the grayscale diffusion of the corresponding image elements, thereby removing the noise points. When_|∇I|>λ_, that is,_|∇I|/λ>1_, the current element is included in the image edge. At this time, as the image gradient continues to increase, the value of the stream function will continue to decrease instead; consequently, the grayscale diffusion at the corresponding image element will continue to weaken until it becomes zero so that the edge information of the brain image can be well preserved.

### Brain Image Processing and Brain Disease Diagnosis Prediction Model Based on Fuzzy Clustering and HPU-Net

**FIGURE 4:**
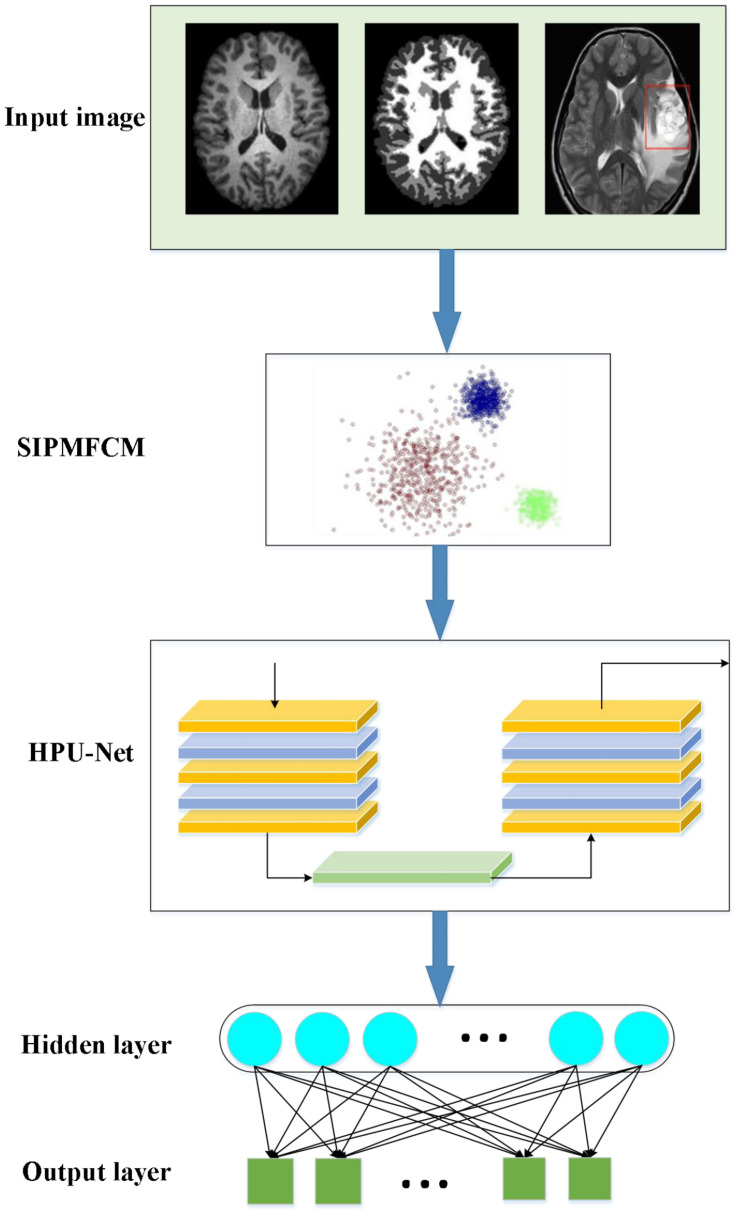
A demonstration to the brain image processing and brain disease diagnosis prediction model based on fuzzy clustering and HPU-Net.

The imaging mechanism of NMR and the complexity of human brain tissues make MRI images present various degrees of noise, weak boundaries, and artifacts. Therefore, the fuzzy clustering algorithm is improved to extract the features of data obtained in the brain image. Afterward, a brain image processing and brain disease diagnosis prediction model based on improved fuzzy clustering and HPU-Net is designed while ensuring the safety performance of the model. Figure 4 illustrates its structure.

In the proposed algorithm, an improved fuzzy clustering algorithm is proposed based on the kernel distance metric regarding the strong noise interference of medical images. This algorithm combines the kernel function, membership constraints, and regularization parameter_ρ_ based on the traditional fuzzy clustering algorithm, making full use of the local spatial information of the image. This makes the algorithm possess better segmentation accuracy and detail retention ability interfered by high-intensity noise, and the image edges after segmentation have better smoothness. This algorithm is recorded as SIPMFCM (Fuzzy Clustering based on Spatial Information Fusion PM) model, described as Equation (25):

(25) $$\begin{array}{l} J_{SIPMFCM}(U,\nu_{1},...\nu_{c},\lambda_{1},...\lambda_{n})\\ =2\sum_{i=1}^{c}\sum_{j=1}^{n}u_{ij}^{m}(1-||x_{j}-v_{i}||)\\ +\sum_{i=1}^{c}\rho_{i}\sum_{j=1}^{n}u_{ij}(1-u_{ij}^{m-1})+2\sum_{i=1}^{c}\rho_{i}\sum_{j=1}^{n}(1-||{\bar{x}}_{j}-v_{i}||) \end{array}$$

In (25),_$\bar{x}$_ refers to the mean filtering brain image or median filtering brain image. The first item here is that the traditional FCM expression is transformed from the kernel distance measurement, which maps the samples to a high-dimensional space and increases the difference between the samples. The second membership penalty item makes the fuzzy division more distinct. The third item here is the neighborhood space restriction item, aiming to strengthen the algorithm’s robustness to image noises. Eventually, the spatial function is integrated into the membership function_*u_ij_*_:

(26) $$u_{i\,j}^{\prime }=\frac{u_{i\,j}^{p}\,s_{i\,j}^{q}}{\sum_{k=1}^{c}u_{i\,k}^{p}\,s_{i\,k}^{q}}$$

**FIGURE 5:**
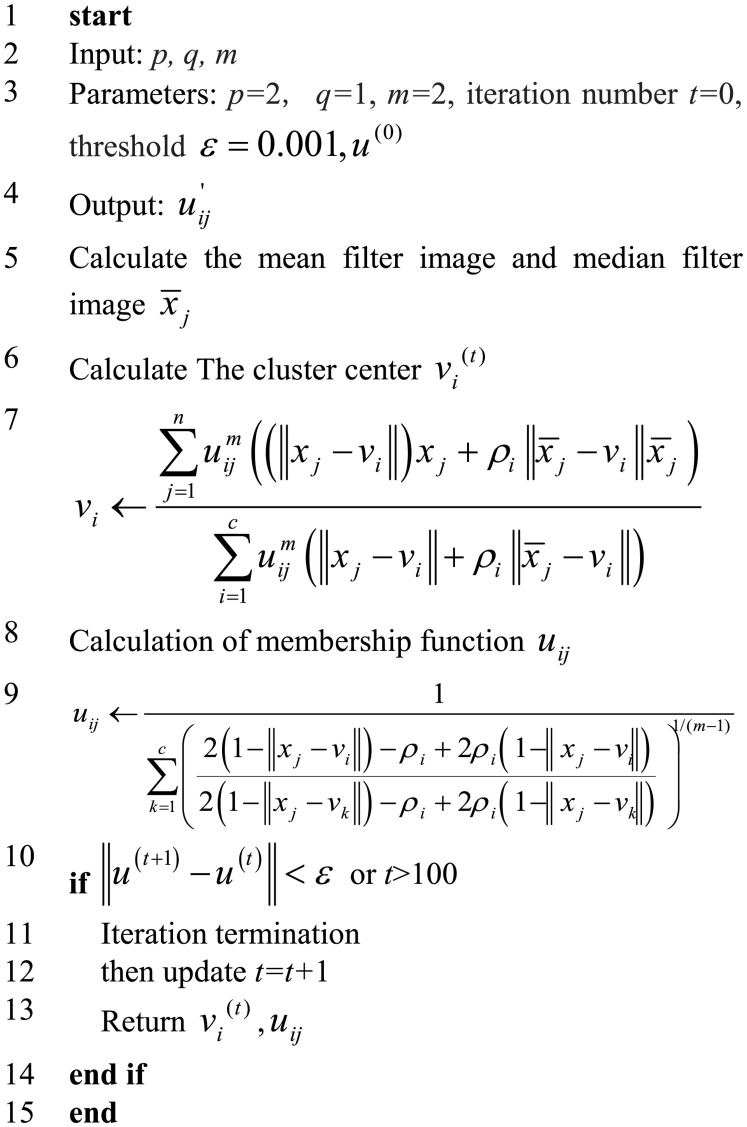
Flowchart of the improved fuzzy clustering algorithm SIPMFCM.

In (26),_*s_ij_*_ is similar to the membership function, representing the possibility that a pixel belongs to a category, and parameters *p* and *q* are the weight parameters that control the membership function and the spatial relationship function, respectively. Here, $u_{i\,j}^{\prime }$ is the final membership function. Figure 5 demonstrates the flowchart of the improved fuzzy clustering algorithm SIPMFCM.

Furthermore, the brain image features are extracted. The U-Net algorithm only uses the features of the last convolutional layer, the information is simple, and it is not enough to contain rich detailed information. Hence, the following improvements are made. First, the introduction of the feature fusion allows the model to fuse information of multiple scales to assist in the image feature extraction. Second, fusing features of multiple scales helps to fuse semantic and location information, complements multiple feature information, and improves the accuracy of feature extraction. Consequently, HPU-Net can process multi-modal MR brain images. Besides, information of different scales is integrated to achieve effective and fast image segmentation.

Each Block in HPU-Net introduces BN (Batch Normalization). This neural network aims to learn the data distribution. Parameters are adopted to simulate data distribution to describe the original data and predict the unknown data (Wang J.L. et al., 2020). During training, the mean value _μβ_ of the internal data distribution of each batch is calculated first as Equation (27):

(27) $$\mu_{\beta }=\frac{1}{m}\,\sum_{i=1}^{m}x_{i}$$

In (27), *m* refers to the number of batches of the brain images. Then, the data distribution variance within each batch_$\delta_{\beta }^{2}$_ is calculated as Equation (28):

(28) $$\delta_{\beta }^{2}=\frac{1}{m}\,\sum_{i=1}^{m}{\left(x_{i}-\mu_{\beta }\right)}^{2}$$

Next, data in the current batch are normalized as Equation (29):

(29) $${\hat{x}}_{i}=\frac{x_{i}-\mu_{\beta }}{\sqrt{\delta_{\beta }^{2}+\varepsilon }}$$

BN can keep the feature with a mean value of 0 and a variance of 1, which is consistent with the input data distribution. Finally, BN also adds a step, “scale and shift,” as described in Equation (30):

(30) $$y_{i}=\gamma \,{\hat{x}}_{i}+\beta =B\,N_{\gamma ,\beta }\,\left(x_{i}\right)$$

Equation (30) seems to be somewhat contrary to the previous operation; however, it can restore the original input. Besides, parameters_γ,β_ shall meet Equation (31):

(31) $$\left\{ \begin{array}{c} \gamma^{\left(k\right)}=\sqrt{V\,a\,r\,\left(x^{\left(k\right)}\right)}\\ \beta^{\left(k\right)}=E\,\left(x^{\left(k\right)}\right) \end{array}\right.$$

In this way, the entire model may or may not change the input distribution of the current layer in the end. The overall effect is to maintain the consistency of the feature distribution to stabilize the model during training and learning. Meanwhile, BN also helps accelerating the convergence time during model training, prevents gradient dispersion or gradient explosion, and improves training accuracy.

### Simulation Experiment

MATLAB is adopted for simulation experiments to verify the performance of the constructed brain image processing and brain disease diagnosis prediction model based on improved fuzzy clustering and HPU-Net. The brain image data obtained in the experiment were all from a Hospital. Data of each patient is standardized to zero mean and unit standard deviation; then, slices that do not contain tumor information are deleted. The image size is cropped to 160 × 160 to reduce data imbalance. To validate the model’s validity, data are divided into five parts equally; then, fivefold cross-validation is performed; that is, four sets of data are used for training and the remaining set of data is used for testing to obtain a set of experimental results. By analogy, different sets of data are adopted to make a test set separately so that five sets of experimental results can be obtained. The average of the five sets of experimental results is taken as the eventual result. The following hyperparameters shall be set for the neural network: 80 iterations, 3,500 rounds of simulations, and 128 batch size. In the performance analysis, the algorithm model proposed is compared with the algorithms applied by other scholars in related fields, CNN (Wu and Cao, 2021), RNN (Roy and Maji, 2020), FCM, LDCFCM (Local Density Clustering Fuzzy C-Means) (Dogra et al., 2020), and AFCM (Adaptive Fuzzy C-Means) (Song et al., 2018) algorithms are selected, respectively, for comparative analysis regarding node survival number, total energy consumption, and residual energy variance. In this simulation, the deep learning algorithm library Keras is utilized, which is an advanced neural network API written in Python with Tensorflow as the back end. Adam is utilized as the optimizer; the standard backpropagation is adopted to train the model, and all parameters are initialized randomly. The computer configuration is Ubuntu 16.04, the CPU (Central Processing Unit) is ES-2640 2,566 memory, and the GPU (Graphics Processing Unit) is GTX 1080Ti.

In brain image feature extraction, the similarity between the segmentation results and the standard expert segmentation results measures the quality of the segmentation results (Debakla et al., 2019). Segmentation evaluation criteria include DSC (Dice Similarity Coefficient), representing the degree of similarity between the segmentation result of the experiment and the expert’s label, ranging from 0 to 1. The higher the DSC value is, the higher the segmentation similarity is. The Jaccard coefficient and DSC are defined as Equations (32) and (33):

(32) $$D\,S\,C\,\left(T,P\right)=\frac{2\,\left|T\,\wedge \,P\right|}{\left|T\right|+\left|P\right|}$$

(33) $$J\,a\,c\,c\,a\,r\,d\,\left(T,P\right)=\frac{\left|T\,\wedge \,P\right|}{\left|T\vee P\right|}$$

In (32) and (33), *T* represents the original tumor area, and *P* represents the segmentation result of the proposed algorithm. While evaluating the segmentation results of the current model, *P* and *T* need to be comprehensively considered.

The evaluation coefficient_*V_pc_*_ and segmentation entropy_*V_pe_*_ are included for analysis and evaluation (Huang et al., 2020). The idea of this evaluation function is that if the fuzzy clustering effect is better, the membership difference of the same pixel belonging to different categories will be greater, the segmentation coefficient obtained will be larger, and the segmentation entropy will be smaller.

(34) $$V_{p\,c}=\frac{\sum_{i=1}^{n}\sum_{j=1}^{c}u_{i\,j}^{2}}{n}$$

(35) $$V_{p\,e}=\frac{\sum_{i=1}^{n}\sum_{j=1}^{c}\left[u_{i\,j}^{2}\,\log u_{i\,j}\right]}{n}$$

## Results and Discussion

### Clustering Process Feasibility

**FIGURE 6:**
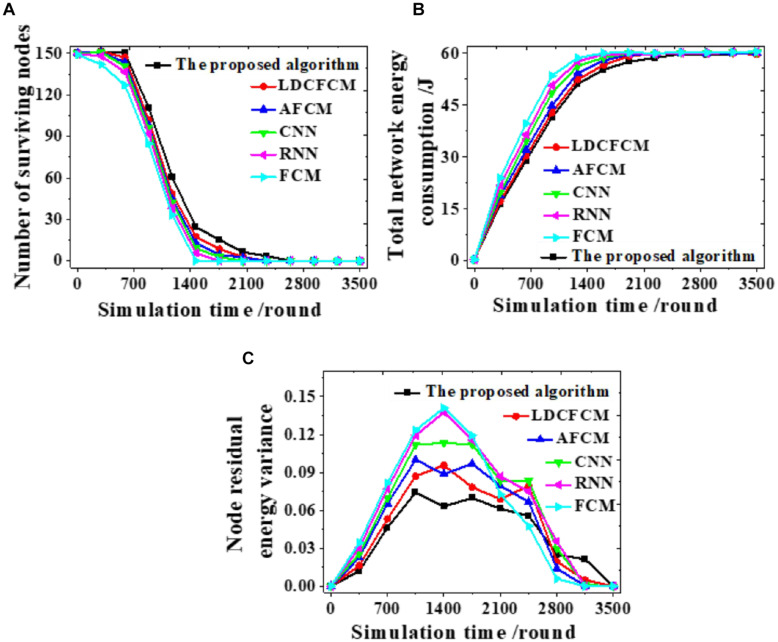
The number of nodes surviving, the total energy consumption of the network, and the node remaining energy variance during the clustering process. **(A)** The number of nodes surviving. **(B)** The total energy consumption of the network. **(C)** The remaining energy variance of the nodes.

Different models are analyzed from the number of nodes surviving, the total energy consumption of the network, and the node remaining energy variance to analyze the feasibility of the proposed algorithm. Figure 6 presents the results.

The clustering process of each model is analyzed. The number of nodes surviving decreases as the number of simulation rounds increases. When the proposed algorithm runs to 2,637 rounds, the number of nodes survived is 0. In comparison, the number of nodes surviving of other models all reduce to 0 at an earlier time (Figure 6A). Changes in remaining energy consumption reveal that the energy consumption of each round of the network is reduced after the algorithm is used for clustering. When the total energy consumption of the network is exhausted, the system simulation reaches 2,560 rounds. In contrast, the total energy consumption of the network of other algorithms is faster, showing that fuzzy clustering reduces the energy consumption of the network (Figure 6B). Moreover, the proposed algorithm fluctuates within 0∼0.070, and the curve changes smoothly. Whereas, the fluctuation range of other models all exceeds 0.070 (Figure 6C). Therefore, the proposed algorithm can provide more nodes survive, lower energy consumption, and more stable changes under the same conditions.

### Overall Network Performance

**FIGURE 7:**
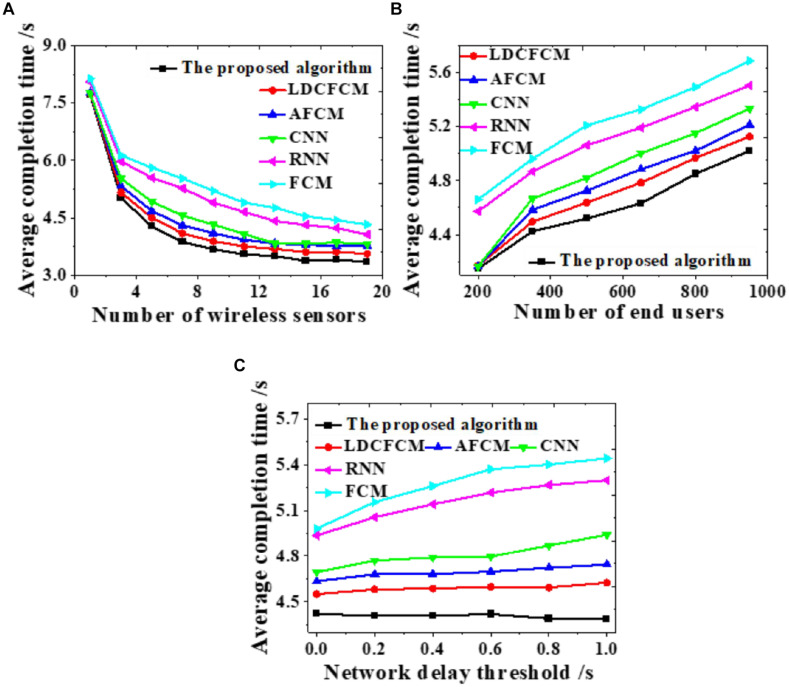
Influences on the average completion time under different factors. **(A)** Number of wireless sensors. **(B)** Number of end-users. **(C)** Network delay threshold.

Influences of the number of wireless sensors, the number of end-users, and changes in network delay threshold on the average task completion time are evaluated to analyze the overall network performance, as displayed in Figure 7.

As the number of wireless sensors increases, the average completion time of the system gradually decreases until it stabilizes (Figure 7A). As the number of end-users increases, the average completion time of system data transmission shows an upward trend (Figure 7B). As the network delay threshold increases, many models do not present notable changes. The average completion time of the proposed algorithm stabilizes at about 4.4 s, while the completion time of RNN and FCM shows an uptrend (Figure 7C). Hence, the proposed algorithm provides better overall network performance than other models all the time, whose average completion time of system data transmission is the shortest.

### Prediction Performance

**FIGURE 8:**
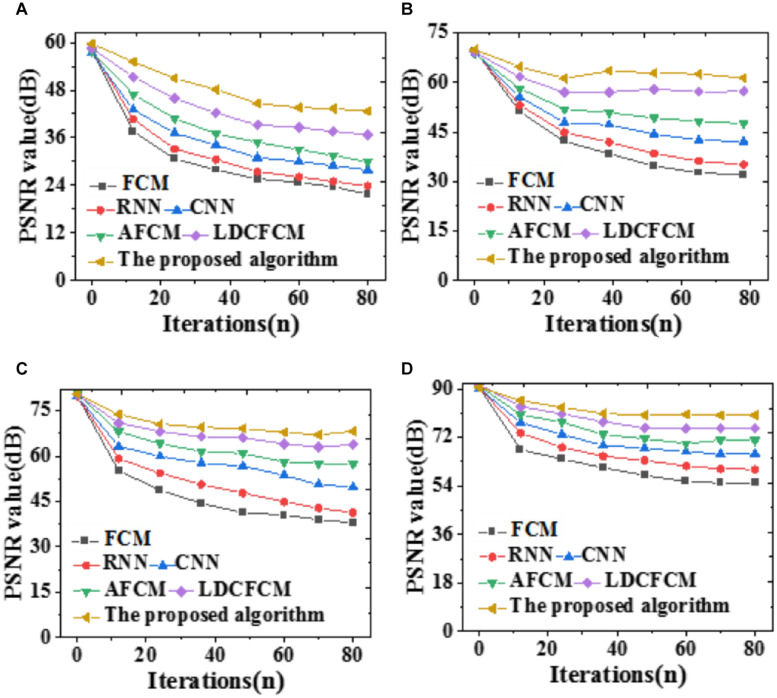
PSNR of the model segmentation results under different brain image noises. **(A)** 10%; **(B)** 20%; **(C)** 30%; **(D)** 40%.

PSNR (Peak Signal-to-Noise Ratio) measures a model’s noise immunity. The larger the PSNR is, the more apparent the denoising effect is, and vice versa. In the present work, all models are compared under 10, 20, 30, and 40% image noises, as in Figure 8.

As the noise value increases, the denoising effect becomes apparent, and the improvement over algorithm PSNR is greater. There are many noise points after segmentation by FCM, which confuses noise and non-noise, resulting in a low PSNR value. Nevertheless, other similar algorithms have a poor removal effect on noise points, and their PSNRs are lower than those of the proposed algorithm. Sometimes, noises in the image seem to be suppressed, and PSNR seems to be improved; however, the edge and detail information of the image will be lost to varying degrees. Next, errors of each model are compared regarding evaluation functions and the Jaccard coefficient. The results are summarized in Tables 1, 2.

**TABLE 1:** Changes in evaluation functions of different models (%).

	**_*V_pc_*_**	**_*V_pe_*_**
The proposed algorithm	0.948	0.051
LDCFCM	0.923	0.069
AFCM	0.864	0.073
CNN	0.847	0.098
RNN	0.803	0.114
FCM	0.795	0.152

**TABLE 2:** Variation of each model with the Jaccard coefficient (%).

	**The white matter**	**The gray matter**
The proposed algorithm	0.854	0.798
LDCFCM	0.839	0.742
AFCM	0.821	0.711
CNN	0.764	0.679
RNN	0.736	0.593
FCM	0.617	0.561

Tables 1, 2 demonstrate that the proposed algorithm provides an evaluation coefficient_*V_pc_*_ of 0.948 and a segmentation entropy_*V_pe_*_ of 0.051, presenting a fuzzy clustering effect significantly better than other models. Regarding the Jaccard coefficient, the proposed algorithm provides recognition accuracies of 0.854 and 0.798 for the white matter and the gray matter on brain images, respectively, apparently better than other algorithms. Therefore, the proposed algorithm provides stronger recognition accuracy and robustness regarding feature recognition and diagnosis prediction.

**FIGURE 9:**
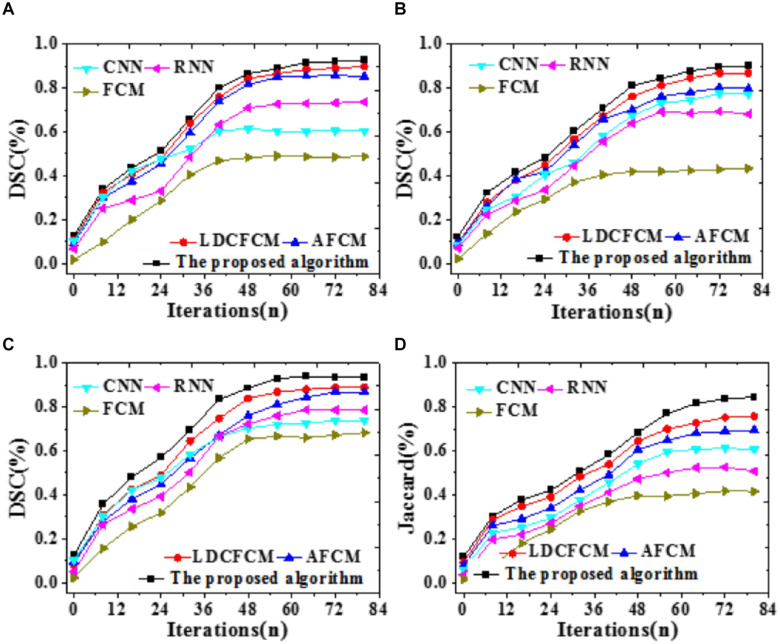
Prediction accuracy indicators of different models for brain images with iterations. **(A)** Core tumor under DSC coefficient. **(B)** Enhancing tumor. **(C)** Whole tumor under DSC coefficient. **(D)** Jaccard coefficient.

To compare the prediction accuracy of different models, DSC is included to predict the recognition effect of the Whole Tumor, Core Tumor, and Enhancing Tumor of brain images; besides, the recognition effect of the Jaccard coefficient to brain images is analyzed, as illustrated in Figure 9.

Figure 9 reveals that DSC and Jaccard coefficient of the proposed algorithm are significantly improved. Under DSC, it provides the highest prediction accuracy for the Whole Tumor, reaching 0.936, followed by Core Tumor and Enhancing Tumor. The segmentation result of the Core Tumor only makes a small improvement under DSC. The Enhancing Tumor only contains the enhanced part of the tumor, occupying a small part of the tumor; sometimes, this area is segmented into other tumor parts. However, the size of the enhanced area is vital in clinical diagnosis. Moreover, the Jaccard coefficient of the proposed algorithm is 0.845, and the segmentation accuracy is remarkably better than that of other algorithms. Therefore, the proposed algorithm based on fuzzy clustering and HPU-Net outperforms other MRI brain tumor image segmentation proposed in recent years.

## Conclusion

Brain tumor images have complicated edge structures and are prone to artifacts and offset fields, affecting image segmentation. The feature extraction and correct diagnosis of brain tumors in multi-sequence MRI images are particularly essential. The traditional fuzzy clustering algorithm is improved to solve the varying degrees of noises, weak boundaries, and artifacts in brain images. Moreover, a brain image processing and brain disease diagnosis prediction model is proposed based on HPU-Net and improved fuzzy clustering. Simulation experiments prove that the proposed algorithm can provide high accuracy for feature extraction and recognition, excellent noise reduction effect, and the optimal image segmentation recognition effect. The highest prediction accuracy for DSC reaches 0.936, and that for the Jaccard coefficient reaches 0.845, providing an experimental basis for brain image feature recognition and brain disease prediction diagnosis. Still, there are some weaknesses in the present work. The proposed algorithm analyzes the overall characteristics while segmenting the brain tumor images, which may cause the difference between different individuals to be insignificant. In the future, texture features and shape features can be utilized for experiments; useful features can be left, and better segmentation results can be obtained by selecting multiple features. Eventually, the key steps to the precise treatment of brain tumors can be implemented to meet the actual needs of clinical medicine, which is vital for the subsequent clinical diagnosis and treatment of brain diseases.

## Data Availability Statement

The raw data supporting the conclusions of this article will be made available by the authors, without undue reservation.

## Author Contributions

MH was mainly responsible for writing the main part of the full text. YZ was responsible for experimental operation and design of related experiments. SX was responsible for data analysis and data processing. HL was responsible for reviewing the full text, verifying the experimental results, and optimizing the structure of the article. ZL was responsible for the collection of relevant statistical data and the verification of the theoretical part. All authors contributed to the article and approved the submitted version.

## Conflict of Interest

The authors declare that the research was conducted in the absence of any commercial or financial relationships that could be construed as a potential conflict of interest.

## Publisher’s Note

All claims expressed in this article are solely those of the authors and do not necessarily represent those of their affiliated organizations, or those of the publisher, the editors and the reviewers. Any product that may be evaluated in this article, or claim that may be made by its manufacturer, is not guaranteed or endorsed by the publisher.
